# Effects of Irradiation on Brain Tumors Using MR-Based Electrical Conductivity Imaging

**DOI:** 10.3390/cancers15010022

**Published:** 2022-12-20

**Authors:** Ji Ae Park, Youngsung Kim, Jiung Yang, Bup Kyung Choi, Nitish Katoch, Seungwoo Park, Young Hoe Hur, Jin Woong Kim, Hyung Joong Kim, Hyun Chul Kim

**Affiliations:** 1 Division of Applied RI, Korea Institute of Radiological and Medical Science, Seoul 01812, Republic of Korea; 2 Office of Strategic R&D Planning (MOTIE), Seoul 06152, Republic of Korea; 3 Medical Science Research Institute, Kyung Hee University Hospital, Seoul 02447, Republic of Korea; 4 Comprehensive Radiation Irradiation Center, Korea Institute of Radiological and Medical Science, Seoul 01812, Republic of Korea; 5 Department of Hepato-Biliary-Pancreas Surgery, Chonnam National University Medical School, Gwangju 61469, Republic of Korea; 6 Department of Radiology, Chosun University Hospital, Gwangju 61453, Republic of Korea

**Keywords:** radiation therapy, electrical conductivity, ionizing radiation, brain tumor, magnetic resonance imaging

## Abstract

**Simple Summary:**

The contrast mechanism of electrical conductivity is preliminarily determined by the concentration and mobility of ions that make up tissues. Recent magnetic resonance-based conductivity imaging has been reported as a highly sensitive tool for measuring and evaluating the responses of normal tissues to irradiation. To evaluate and assess its therapeutic effects in clinical practice, it is required to verify the response of malignant tissues to irradiation. In this study, the responses of tumor tissues following irradiation were quantified and compared with the responses of normal tissues. Conductivity at high frequencies provides information on the changes in cellularity and the amounts of electrolytes inside tumor tissues, showing potential as an imaging tool for quantifying the therapeutic effects of radiation on tumors by measuring absolute values and calculating percentage changes. For clinical applications, the imaging results of large samples and statistical analysis of the relationship between conductivity changes and tissue responses are required.

**Abstract:**

Ionizing radiation delivers sufficient energy inside the human body to create ions, which kills cancerous tissues either by damaging the DNA directly or by creating charged particles that can damage the DNA. Recent magnetic resonance (MR)-based conductivity imaging shows higher sensitivity than other MR techniques for evaluating the responses of normal tissues immediately after irradiation. However, it is still necessary to verify the responses of cancer tissues to irradiation by conductivity imaging for it to become a reliable tool in evaluating therapeutic effects in clinical practice. In this study, we applied MR-based conductivity imaging to mouse brain tumors to evaluate the responses in irradiated and non-irradiated tissues during the peri-irradiation period. Absolute conductivities of brain tissues were measured to quantify the irradiation effects, and the percentage changes were determined to estimate the degree of response. The conductivity of brain tissues with irradiation was higher than that without irradiation for all tissue types. The percentage changes of tumor tissues with irradiation were clearly different than those without irradiation. The measured conductivity and percentage changes between tumor rims and cores to irradiation were clearly distinguished. The contrast of the conductivity images following irradiation may reflect the response to the changes in cellularity and the amounts of electrolytes in tumor tissues.

## 1. Introduction

Radiation therapy (RT) has been used as an effective treatment for cancer [1]. The use of high-energy radiation from X-rays, gamma rays, neutrons, and other sources can kill cancer cells and shrink tumors [2]. RT in tumor tissues aims to maximize the suppression of local tumors and minimize side effects on normal tissues [2,3]. Along with the treatment of malignant brain tumors, it has many advantages in the treatment of functional brain diseases, such as benign brain tumors, trigeminal neuralgia, cerebral tumor vein malformations, and movement disorders [4,5,6,7,8]. Meanwhile, RT in children should be given special attention because radiation tolerance is relatively low in children compared to adult patients. In addition, side effects from RT can last a lifetime [9,10,11].

Image-guided radiation therapy (IGRT) and intensity-modulated radiation therapy (IMRT) are commonly used in clinical practice [12]. In combination with computed tomography (CT) or magnetic resonance imaging (MRI), IGRT makes it possible to accurately estimate irradiated regions [13]. The application of IMRT has enabled sophisticated RT. IGRT generally uses CT images; however, there are several limitations, such as relatively low image resolution and secondary radiation exposure to the patient’s brain during acquisition [14]. In the case of IMRT using X-rays, radiation energy can penetrate normal tissues of the human body. It is continuously absorbed until it reaches tumor tissues [15,16,17]. To prevent acute radiation syndrome, low-energy radiation should be irradiated repeatedly. However, there is a limitation in measuring the standard irradiation dose [18]. Therefore, monitoring the treatment response and quantifying the treatment effect after irradiation are important factors in the assessment of cancer treatment.

The recent magnetic resonance (MR)-based electrical conductivity imaging method has been proposed to be a reliable tool for measuring the tissue response immediately after irradiation [19,20]. The sensitivity of conductivity imaging is superior to conventional MR imaging because its contrast originates from the concentration and mobility of ions in response to irradiation [19]. The measured conductivity and percentage changes are clearly different depending on the amount of irradiation and elapsed time [20]. In addition, the conductivity changes are different depending on the source of irradiation [19,20]. Although conductivity imaging has sufficient sensitivity to distinguish responses of normal tissues to irradiation, application to cancerous tissues is required as a useful tool that can be applied to clinical practice.

Most of the previous results using MR-based electrical conductivity imaging were obtained from normal tissues [19,20]. The measurement and quantification of normal tissue responses to irradiation are useful for determining radiation-induced injury caused by unintentional exposure to ionizing radiation [20]. However, compared to normal tissues, cancerous tissues have different cellular environments regarding the concentration and mobility of ions [21]. In particular, it is known that the conductivity of cancerous tissue is clearly different from that of normal tissue due to differences in cellularity and tissue homogeneity [21,22]. Therefore, in terms of electrical conductivity, the response of cancerous tissue to irradiation is predicted to be different from the response of normal tissue.

The purpose of this study was to non-invasively evaluate tissue response following irradiation in brain tumors based on absolute conductivity changes. MR-based conductivity imaging was performed on brain tumor model mice, which were composed of irradiated and non-irradiated groups. For quantitative analysis, absolute conductivity and percentage change of brain tissues were measured and calculated for the tumor region and the contralateral region in both groups. Time-course variations in the tissue responses of both groups were compared before and up to 10 days post-irradiation. For intra-tissue comparison, the absolute conductivity and percentage changes were obtained at the core and rim of a tumor.

## 2. Materials and Methods

### 2.1. Tumor Cell Culture

C6 glioma cells from the American Type Culture Collection were cultured in growth media consisting of Minimum Essential Medium alpha (Corning, New York, NY, USA) supplemented with 10% (*v*/*v*) fetal bovine serum (JR Scientific Inc., Woodland, CA, USA) and 1% antibiotic-antimycotic (Gibco, Billings, MT, USA). The cells were cultured in a humidified 5% CO_2_ atmosphere at 37 °C.

### 2.2. Animal Preparation for Tumor Model

A total of 14 Balb/c nude mice (male, 6 weeks old, weighing 20~23 g) were used for in vivo imaging experiments. Animal care, maintenance, and treatments were performed according to the protocol approved by the Institutional Animal Care and Use Committee (IACUC, No. 2021-0077) of the Korea Institute of Radiological & Medical Sciences (KIRAMS). Intracranial tumor models were induced in a sterile environment using sterile instruments. The mice were anesthetized by intramuscular injection with Zoletile (Virbac Laboratories, Carros, France), Rompun (Bayer, Leverkusen, Germany), and a saline mixture (*v*/*v*, 5:2:3, 1 μL/g body weight). After fixing the mouse heads in a stereotactic frame, the scalps were cut. The skulls were pierced to the right (1 mm) and anterior (2 mm) of the bregma. For intracranial tumors, C6 glioma cells (1 × 10^6^ cells/5 μL serum-free media) were injected into the right caudate–putamen at a depth of 3 mm using a Hamilton syringe (injection rate: 1 μL/min). At 3 min after injection, the syringe was slowly removed, and the incision was sutured.

### 2.3. Radiation Exposure

After 2 weeks of tumor cell inoculation, tumor growth was confirmed in the MR images using a 9.4T MRI scanner (Agilent Technologies, Santa Clara, CA, USA). The mice were divided into two groups: an irradiated group (*n* = 7) and a non-irradiated group (*n* = 7). In the case of the irradiated group, the mouse heads were positioned in the center of the exposure area. The mean dose rate was 0.98 Gy/min and the field size was 5 × 30 cm under a Co-60 gamma-ray irradiation unit (Gamma Beam 100-80, 780, Best Theratronics, Kanata, ON, Canada). The distance between the source of radiation and the head skin was 80 cm. During irradiation, each mouse was maintained under anesthesia by mixing Zoletile and Rompun. Each mouse was housed in an individual cage for the imaging experiments.

### 2.4. Imaging Experiments

**Figure 1:**
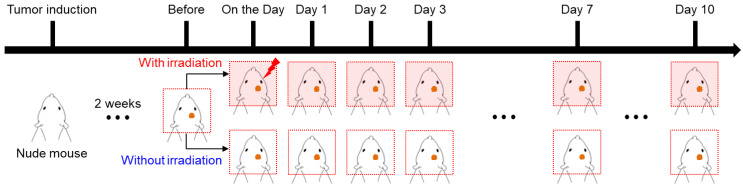
Schematic illustration of the experimental setup for MR-based electrical conductivity imaging to measure the effects of irradiation on in vivo mouse brain tumors.

All 14 mice were subjected to imaging experiments before and at 0, 1, 2, 3, 7, and 10 days after irradiation (Figure 1). The imaging experiments were also performed on the non-irradiated group during the same time period. After anesthetizing mice with isoflurane (anesthetized with a 1–2.5% isoflurane in oxygen), the mice were placed inside the bore of an MRI scanner. The MR and electrical conductivity images of the mouse brains were obtained from an MRI scanner with a birdcage RF coil. For the MR images, a fast spin-echo multi-slice (FSE-MS) pulse sequence was applied with the following imaging parameters: repetition time (TR) = 3500 ms, echo time (TE) = 30 ms, echo train length (ETL) = 6, number of averaging = 2, slice thickness = 1 mm, number of slices = 5, matrix size = 192 × 192, field-of-view (FOV) = 50 × 50 mm^2^, and total imaging time = 2 min 48 s. For the electrical conductivity imaging, a multi-echo multi-slice (MEMS) spin-echo pulse sequence was applied to obtain a B1 map, which was used to calculate high-frequency conductivity images. The imaging parameters were: TR = 2200 ms, TE = 22 to 132 ms, number of echoes = 6, number of averaging steps = 5, slice thickness = 1 mm, number of slices = 5, matrix size = 128 × 128, FOV = 50 × 50 mm^2^, and total imaging time = 23 min 46 s. To obtain the apparent diffusion coefficient (ADC), diffusion-weighted imaging (DWI) was performed using a gradient-echo echo planar imaging (GR-EPI) pulse sequence. The imaging parameters were: TR = 2000 ms, TE = 23.3 ms, number of b-values = 0 and 1000 s/mm^2^, number of averaging steps = 1, slice thickness = 1 mm (no gap), number of slices = 15, matrix size = 128 × 128, FOV = 50 × 50 mm^2^, and total imaging time = 8 min 32 s.

### 2.5. Conductivity Measurement and Analysis

MR imaging was used to confirm the morphological changes of the mouse brains before and after irradiation in both groups. Electrical conductivity was reconstructed from the optimized B1 phase images obtained from the MEMS data after multiple pre-processing steps, as follows. The raw data were extracted from the k-space of the MR spectrometer. The MEMS images were reconstructed by 2D fast Fourier transform and then separated into magnitude and phase images. The phase images were unwrapped using the PUMA algorithm [23], and the unwrapped phase images of each echo were averaged to achieve a higher signal-to-noise ratio (SNR) using a weighting factor. The details of the conductivity image reconstruction process followed the work of Katoch et al. [21,23].

Since electrical conductivity is a material property that provides an absolute value, we measured the conductivity values for the regions-of-interests (ROIs) in both groups. The ROIs were positioned to cover the same areas of tumor tissues and contralateral regions and were also located at the cores and rims of the tumors for intra-tissue comparison. The absolute conductivity of the mouse brain tissues was used to quantify the irradiation effects. The percentage change (%), which indicates the degree of response depending on irradiation, was calculated following the irradiation based on the values before irradiation. Time-course variations in the tissue response of both groups were compared before and up to 10 days post-irradiation. Statistical comparisons were conducted using SPSS software (v. 20.0 for Windows; SPSS IBM, Armonk, NY, USA). The significant differences in conductivity with and without irradiation in the ROI were compared using a two-sample *t*-test.

## 3. Results

### 3.1. Conductivity Images of Brain Tumors with and without Irradiation

**Figure 2:**
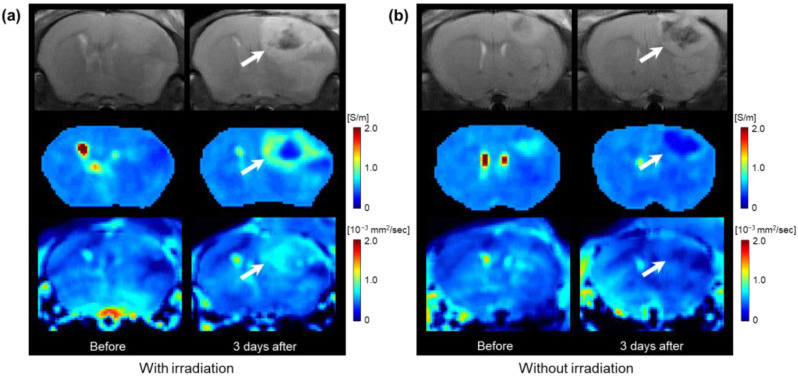
In vivo MR (upper), electrical conductivity (middle), and ADC (lower) images of C6 glioma model mouse brains regarding tissue responses with (**a**) and without (**b**) irradiation. All images were acquired before and 3 days after irradiation. The growth of C6 glioma with central necrosis (white arrows) was observed in the left hemisphere.

Figure 2 shows the MR, electrical conductivity, and ADC images of the in vivo mouse brain tumors with and without irradiation. All images were acquired for the irradiated (Figure 2a) and non-irradiated (Figure 2b) groups before and 3 days after irradiation. In the MR images, the morphological changes of the tumor regions were observed in both groups compared to those before irradiation. There was no clear difference in the tumor regions 3 days after irradiation between the two groups. On the contrary, the conductivity images show marked changes in the irradiated group compared to the non-irradiated group. Specifically, the conductivity of the tumor rims showed increased contrast in the irradiated group compared to the non-irradiated group. The ADC maps show a similar pattern to that of the conductivity, but the contrast change was relatively low.

**Figure 3:**
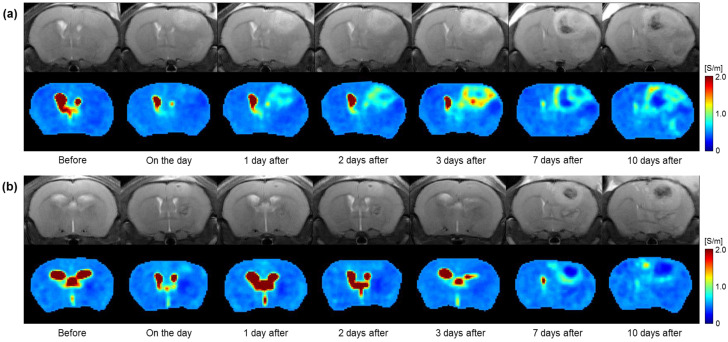
Time-course variations in MR (upper) and electrical conductivity (lower) images of in vivo mouse brains in the group with (**a**) and without (**b**) irradiation.

Figure 3 shows the full time-course images of the MR and electrical conductivity of the in vivo brain tumors with and without irradiation. All images were obtained before and 0, 1, 2, 3, 7, and 10 days after in the irradiated (Figure 3a) and non-irradiated (Figure 3b) groups. Compared to before irradiation, the morphological changes in the tumor region in the MR images show a similar pattern over time in both groups. However, the conductivity images show clear contrast changes between the two groups over time. Specifically, the conductivity of the irradiated group shows an increase up to 3 days after and a slight decrease up to 10 days. On the contrary, the conductivity of the non-irradiated group shows similar contrast up to 3 days and changes up to 10 days. The contrast of the tumor rims was clearly different between the two groups.

### 3.2. Quantification of Irradiation Effects in Brain Tumors

**Figure 4:**
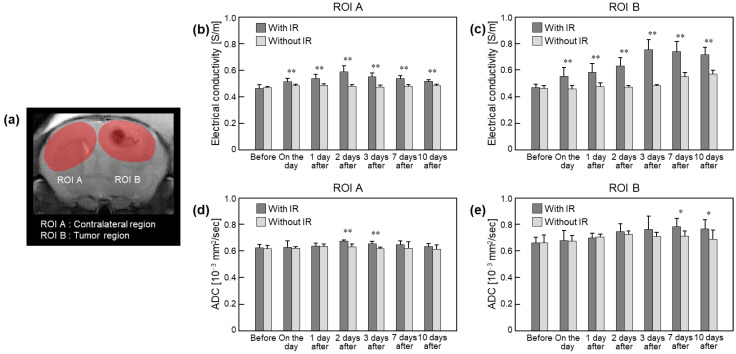
Comparison of absolute conductivity and apparent diffusion coefficient (ADC) in brain tissues with and without irradiation. ROIs (**a**) were located in the contralateral and tumor regions at full time points. Bar graphs indicate measured absolute conductivity (**b**,**c**) and corresponding ADC (**d**,**e**) values at ROIs before and after irradiation. Statistical significances were compared between irradiated and non-irradiated groups (* *p* < 0.05, ** *p* < 0.01).

Figure 4 and Table 1 show a comparison of the absolute conductivity from in vivo mouse brain tissues with and without irradiation. The ROIs were placed to cover all tumor tissues and the contralateral regions in the same area (Figure 4a). The conductivity of the irradiated tissue was higher than that of the non-irradiated tissue in both ROIs (Figure 4b,c). Specifically, the conductivity of the contralateral region with irradiation showed an increase up to 2 days after and a gradual decrease up to 10 days (Figure 4b). There were no clear changes in the tissues without irradiation. On the contrary, the conductivity of the tumor regions with irradiation showed increases up to 3 days after and slight decreases up to 10 days (Figure 4c). The conductivity of the tumor regions without irradiation showed similar contrast up to 3 days after and gradually increased up to 10 days. The ADCs in the ROIs showed a similar pattern as the changes in conductivity, but the changes were relatively small.

**Figure 5:**
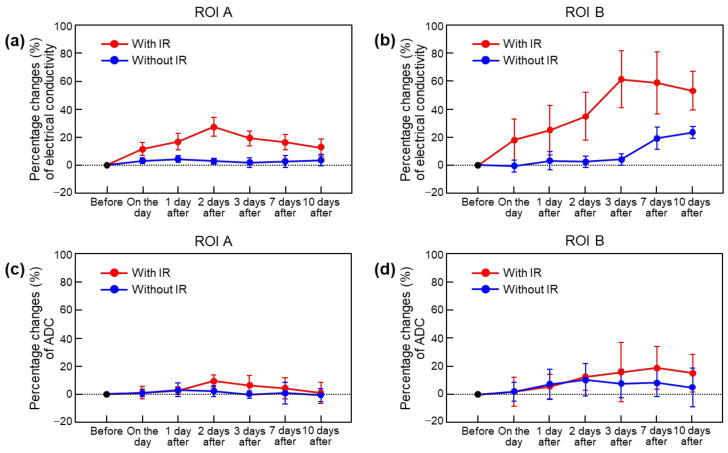
Comparison of percentage changes in absolute conductivity and apparent diffusion coefficient (ADC) with and without irradiation. Percentage changes in electrical conductivity (**a**,**b**) and ADC (**c**,**d**) in ROIs were calculated following irradiation based on values before irradiation.

Figure 5 and Table 1 (in parentheses) show comparisons of the percentage changes in the in vivo mouse brain tissues with and without irradiation. The percentage changes of the contralateral region with irradiation increased by 27.2% up to 2 days and then decreased by 12.4% (Figure 5a). Percentage changes of the contralateral region without irradiation were not observed. Meanwhile, the percentage changes of the tumor regions with irradiation increased by 61.1% up to 3 days and then decreased by 52.9% (Figure 5b). Percentage changes of the tumor regions without irradiation were not observed until 3 days after irradiation but increased to 23.2% 10 days after. On the contrary, the percentage changes in the ADCs were within a maximum of 10% in the contralateral region (Figure 5c) and within a maximum of 20% in the tumor region (Figure 5d).

**Figure 6:**
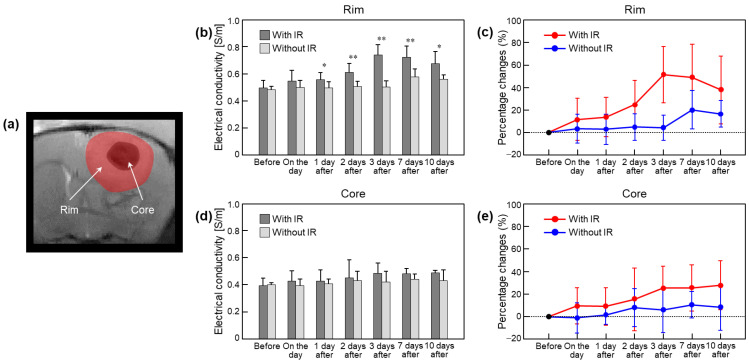
Comparison of absolute conductivity and percentage changes in mouse brain tumor tissues with and without irradiation. ROIs (**a**) were located in the rims and cores of tumor regions at full time points. Bar graph (**b**,**d**) indicates measured absolute conductivity in tumor tissues after irradiation. Line graph (**c**,**e**) indicates percentage changes of conductivity in tumor tissues after irradiation. Statistical significances were compared between irradiated and non-irradiated groups (* *p* < 0.05, ** *p* < 0.01).

Figure 6 shows a comparison of the absolute conductivity and percentage changes focusing on brain tumor tissues with and without irradiation. ROIs were placed on the rims and cores of tumors (Figure 6a). The conductivity of the tumor rims with irradiation showed an increase up to 3 days and a slight decrease up to 10 days (Figure 6b). The conductivity of the tumor rims without irradiation showed similar contrast up to 3 days and then changed. The percentage changes of the tumor rims with irradiation increased by 51.2% up to 3 days after irradiation and then decreased by 37.6% (Figure 6c and Table 1). Percentage changes in the tumor rims without irradiation were not observed until 3 days after irradiation but changed by 16.5% 10 days after. On the contrary, the conductivity of the tumor cores showed a gradual increase after irradiation in both groups, but the changes were relatively large in the irradiated group (Figure 6d). The percentage changes of the tumor cores were within a maximum of 27.8% in the irradiated group and within a maximum of 10.6% in the non-irradiated group (Figure 6e and Table 1).

## 4. Discussion

The contrast of MR-based electrical conductivity images is primarily determined by the concentration and mobility of ions constituting the tissue [19,20,21,22,23]. The effect of a change in the ion concentration is independent of the frequency, whereas the effect of a change in the mobility is dependent on the frequency because ion movements are hindered by cell membranes at low frequencies [21]. The conductivity at high frequencies provides mixed information about the extracellular- and intracellular compartments, whereas the conductivity at low frequencies provides mostly ionic information about the extracellular compartment [22,23]. Radiation therapy (RT) is a treatment method using ionizing radiation, which is generally provided as part of cancer therapy to control or kill malignant cells [2,3,4]. Several studies have reported that electrical conductivity images at high frequencies show advantages in measuring and evaluating tissue responses following ionizing radiation [19,20]. In this study, we used a 9.4T MRI with a fixed frequency of 400 MHz. The conductivity contrast was mostly due to differences in the ion concentrations in different tissues.

Cancer can be defined as an uncontrollable rate of cell growth, increased proliferation, and a decrease in cell death, which can lead to tumor formation [2]. According to tumor growth, the volume of the tumor is increased and the total amounts of the cellular components within tumor tissues are eventually increased [24]. The high cellularity of the tumor indicates that the proportion of the intracellular space within the tumor tissue is relatively high compared to that of the extracellular space [2,3,24,25]. Ion channels play a critical role in tumors regarding cell proliferation, malignant angiogenesis, migration, and metastasis [25]. The amounts of positive and negative ions in the intracellular space are larger than those of the extracellular space [24,25]. Thus, the total amount of ions in tumors will be increased with an increase in tumor cellularity. In this study, the conductivity changes of tumor tissues without irradiation may have supported the above phenomenon during tumor growth. Meanwhile, the conductivity of the contralateral region without irradiation was not changed because there was no significant change in the total amounts of positive and negative ions due to constant cellularity and the amount of interstitial fluid.

Conductivity changes caused by irradiation in the tumor tissues were clearly distinguished from those in the tumor tissue without irradiation. The main events of irradiation effects in the intracellular space include damage to the cellular deoxyribonucleic acid, the generation of reactive oxygen species, and the generation of reactive nitrogen species after irradiation [26]. These will eventually result in cell death, such as apoptosis, necrosis, or autophagy [26]. The timing of radiation-induced injury depends on the life span of mature cells, and the expression of radiation cell death is generally delayed until mitosis [26]. Muscle and brain cells are known to be relatively insensitive to radiation [26]. In general, normal brain tissue is more resistant to radiation compared to brain tumors. Glial tumor cells are especially more susceptible to radiation than normal brain tissues. RT is considered the treatment of choice for gliomas due to the radiosensitivity of gliomas [27,28]. Mixed with the pathological changes of tumor growth, the increased conductivity in the tumor tissues after irradiation can be inferred from this. However, the amount of irradiation was not sufficient enough for immediate cell death in this study. Mitosis was not active in the brain tissues. The time point of 10 days after irradiation was too early to observe extensive cell death. In addition, considering that the central necrosis of tumors was observed even in the non-irradiated group, it is thought that the tumor necrosis was mainly due to hypoxia and nutrient deficiency caused by tumor growth.

Conductivity changes in the contralateral region with irradiation showed the same pattern as those reported in previous studies [19,20]. Although the conductivity changes might be different depending on the irradiation method, it should be noted that the pattern over time after irradiation was similar to previous results obtained from normal brain tissues [20]. Interestingly, the timing of conductivity changes and residual contrast by irradiation was different between tumor and contralateral brain tissues. Compared to before irradiation, the percentage changes of the tumor regions increased by 61.1% up to 3 days and then decreased by 52.9%. The percentage changes of the contralateral regions showed an increase of 27.2% up to 2 days and then decreased by 12.4%. Although there is no direct evidence for this result, we can infer that it was related to the increase in the cellularity according to the malignancy of the tumor 3 days after irradiation. Specifically, as the malignant degree of a tumor increases, the degree of tumoral cellularity also tends to increase in glioma [2,24,25,26,27,28,29]. However, this should be dealt with in future studies.

During tumor growth, necrosis is common in the core of the tumor due to hypoxia from insufficient vascularization and subsequent metabolic stresses, such as nutrient deficiency [30]. When the necrotic cell membrane breaks down, cytoplasmic components, including electrolytes are released into the extracellular space through the damaged membrane [24,31]. As a result, cell necrosis finally promotes delayed inflammatory responses, and the total electrolytes slightly increase [31,32]. In this study, the conductivity of the tumor cores slightly increased in the non-irradiated group. This coincided with the timing of the appearance of core regions considered to be central coagulation necrosis in the MR images. The conductivity difference in the cores of tumors between the irradiated and non-irradiated groups may be related to the ionization of the electrolytes by irradiation. This can be inferred from the result of Kim et al. [20], which showed that the measured conductivity following irradiation was different depending on the type of solutions and irradiation dose used.

From the results of Kim et al. [20], the conductivity increased more in the saline solution than in distilled water after irradiation because the total amount of electrolytes was higher in the saline solution than in distilled water. Since the amounts of ions in the intracellular space are larger than those of the extracellular space [2,3,24,25], the tumor rims can be considered to contain the electrolytes of a viable tumor with higher cellularity, while tumor cores are considered to be extracellular spaces due to tumor necrosis. ROI A was considered to contain electrolytes of normal brains with lower cellularity. Therefore, the total amount of electrolytes was considered to be the highest in the order of tumor rims, normal brains, and tumor cores. In this study, the absolute conductivity was the highest in the order of tumor rims, normal brains, and tumor cores in both the irradiated and non-irradiated groups. The difference in the conductivity according to irradiation and tissue types was mainly due to the difference in electrolyte amount caused by the cellularity.

There are several limitations to this study. First, there were no histopathological findings to support the imaging results. A correlation between the imaging results and histopathological findings could better differentiate the RT-induced tissue damage among the viable tumor cells, tumor necrosis, and normal brains by providing detailed information on cellularity [33]. Second, this study was focused on the feasibility of conductivity imaging for the acute response of brain tumor models after irradiation. To determine lethal or sublethal tissue damage for clinical significance, long-term conductivity imaging for early and late delayed responses should be performed. Third, partial extracerebral tumor formation in some models might influence the conductivity of intracellular tumors through the partial absorption of irradiation. However, even if there was some influence, we believe it would not significantly change the tendency of differences in the conductivity in the tumor rims, normal brains, and tumor cores. Finally, together with imaging data from a large sample size, statistical analysis between the conductivity changes and tissue response should be performed at more time points for clinical applications.

## 5. Conclusions

The goal of radiation therapy in cancers is to maximize the suppression of local tumors and minimize the side effects on normal tissue response by irradiation progress slowly over time. MR-based electrical conductivity imaging showed potential as a tool with high sensitivity for measuring and evaluating tissue response after irradiation. We applied this technique to tumors to quantify the responses of tumor tissues following irradiation and to evaluate the responses of normal tissues. The high-frequency conductivity images effectively showed an acute response after irradiation in glial tumors. The high-frequency conductivity images can differentiate brain tissues, including viable tumors, tumor necrosis, and normal brains. MR-based electrical conductivity shows potential as a tool to differentiate the therapeutic effect of radiation by measuring the absolute values and calculating the percentage changes. Future studies should focus on the conductivity imaging of tumor patients following sufficient validation, including long-term time points that cover early and late delayed response as well as the differentiation of tumoral cellularity.

## Figures and Tables

**Table 1:** Summary of absolute conductivity and percentage changes (in parentheses) obtained from in vivo brain tumor conductivity images. Conductivity values indicate tissue response to irradiation at a full time-course dataset. Percentage changes indicate the degree of tissue response to irradiation based on values pre-irradiation.

In Vivo Brain		Electrical Conductivity (S/m) (Percentage Change, %)						
		Before	On the Day	1 Day After	2 Days After	3 Days After	7 Days After	10 Days After
ROI A	With IR	0.462 ± 0.029 (-)	0.514 ± 0.022 (11.4 ± 4.9)	0.538 ± 0.030 (16.6 ± 5.9)	0.588 ± 0.044 (27.2 ± 6.7)	0.550 ± 0.030 (19.1 ± 5.3)	0.536 ± 0.021 (16.3 ± 5.6)	0.515 ± 0.013 (12.4 ± 6.1)
	Without IR	0.467 ± 0.010 (-)	0.482 ± 0.014 (3.0 ± 1.9)	0.487 ± 0.010 (4.2 ± 2.4)	0.480 ± 0.009 (2.8 ± 1.9)	0.473 ± 0.013 (1.7 ± 3.4)	0.479 ± 0.012 (2.6 ± 4.3)	0.483 ± 0.012 (3.5 ± 4.0)
ROI B	With IR	0.469 ± 0.024 (-)	0.552 ± 0.066 (18.0 ± 14.6)	0.582 ± 0.067 (24.8 ± 17.6)	0.629 ± 0.064 (34.7 ± 17.1)	0.752 ± 0.075 (61.1 ± 20.4)	0.739 ± 0.075 (58.4 ± 22.2)	0.714 ± 0.056 (52.9 ± 13.8)
	Without IR	0.463 ± 0.020 (-)	0.459 ± 0.024 (−0.7 ± 4.3)	0.476 ± 0.026 (3.0 ± 6.5)	0.473 ± 0.012 (2.3 ± 4.2)	0.482 ± 0.008 (4.0 ± 4.1)	0.550 ± 0.030 (19.1 ± 8.0)	0.570 ± 0.029 (23.2 ± 4.4)
Rim	With IR	0.496 ± 0.057 (-)	0.547 ± 0.077 (11.6 ± 18.6)	0.556 ± 0.053 (13.7 ± 17.3)	0.610 ± 0.067 (24.9 ± 21.2)	0.738 ± 0.077 (51.2 ± 25.0)	0.723 ± 0.080 (48.8 ± 29.3)	0.674 ± 0.091 (37.6 ± 29.9)
	Without IR	0.483 ± 0.024 (-)	0.498 ± 0.053 (3.4 ± 12.6)	0.494 ± 0.048 (2.9 ± 13.4)	0.504 ± 0.042 (4.9 ± 11.6)	0.501 ± 0.046 (4.1 ± 11.2)	0.576 ± 0.058 (20.1 ± 16.9)	0.560 ± 0.034 (16.5 ± 11.8)
Core	With IR	0.392 ± 0.058 (-)	0.427 ± 0.075 (9.7 ± 15.9)	0.426 ± 0.084 (9.2 ± 16.5)	0.452 ± 0.131 (15.4 ± 27.7)	0.485 ± 0.076 (25.4 ± 19.5)	0.481 ± 0.039 (25.5 ± 20.4)	0.488 ± 0.020 (27.8 ± 21.6)
	Without IR	0.399 ± 0.015 (-)	0.394 ± 0.049 (−1.0 ± 13.5)	0.405 ± 0.038 (1.6 ± 8.4)	0.432 ± 0.069 (8.1 ± 16.7)	0.422 ± 0.076 (6.1 ± 20.1)	0.440 ± 0.040 (10.6 ± 11.7)	0.432 ± 0.079 (8.4 ± 20.3)

## Data Availability

The data presented in this study are available upon request from the corresponding author. The data are not publicly available for confidentiality reasons.
